# A metabolite-based liquid biopsy for detection of ovarian cancer

**DOI:** 10.1186/s40364-024-00629-2

**Published:** 2024-08-28

**Authors:** Johannes F. Fahrmann, Seyyed Mahmood Ghasemi, Chae Y. Han, Ranran Wu, Jennifer B. Dennison, Jody Vykoukal, Joseph Celestino, Karen Lu, Zhen Lu, Charles Drescher, Kim-Anh Do, Samir Hanash, Robert C. Bast, Ehsan Irajizad

**Affiliations:** 1 Department of Clinical Cancer Prevention, The University of Texas M. D. Anderson Cancer Center, Houston, TX USA; 2 Department of Biostatistics, The University of Texas M.D. Anderson Cancer Center, 6767 Bertner Street, Houston, TX 77030 USA; 3 Department of Experimental Therapeutics, The University of Texas M. D. Anderson Cancer Center, Houston, TX 77030 USA; 4 Department of Gynecological Oncology and Reproductive Medicine, The University of Texas M. D. Anderson Cancer Center, Houston, TX 77030 USA; 5 Translational Research Program, Fred Hutchinson Cancer Research Center, Seattle, WA USA; 6 Division of Gynecologic Oncology, Swedish Cancer Institute, Seattle, WA USA

**Keywords:** Metabolites, Biomarkers, Early detection, Ovarian cancer

## Abstract

Serial CA125 and second line transvaginal ultrasound (TVS) screening in the UKCTOCS indicated a shift towards detection of earlier stage ovarian cancer (OvCa), but did not yield a significant mortality reduction. There remains a need to establish additional biomarkers that can complement CA125 for even earlier and at a larger proportion of new cases. Using a cohort of plasma samples from 219 OvCa cases (59 stage I/II and 160 stage III/IV) and 409 female controls and a novel Sensitivity Maximization At A Given Specificity (SMAGS) method, we developed a blood-based metabolite-based test consisting of 7 metabolites together with CA125 for detection of OvCa. At a 98.5% specificity cutpoint, the metabolite test achieved sensitivity of 86.2% for detection of early-stage OvCa and was able to capture 64% of the cases with low CA125 levels (< 35 units/mL). In an independent test consisting of 65 early-stage OvCa cases and 141 female controls, the metabolite panel achieved sensitivity of 73.8% at a 91.4% specificity and captured 13 (44.8%) out of 29 early-stage cases with CA125 levels < 35 units/mL. The metabolite test has utility for ovarian cancer screening, capable of improving upon CA125 for detection of early-stage disease.

**Supplementary Information:**

The online version contains supplementary material available at 10.1186/s40364-024-00629-2.

To the editor

Currently, over 70% of patients with ovarian cancer present with advanced stage (III-IV) disease, which contributes to dismal long-term survival rates of less than 30%. Five-year survival rates up to 70–90% can be achieved with conventional surgery and chemotherapy, when disease is localized to the ovary (stage I) or pelvis (stage II) [1, 2]. A two-stage strategy using the Risk of Ovarian Cancer Algorithm (ROCA) whereby rising CA125 prompts transvaginal ultrasound (TVS) has been applied for screening and shown to achieve adequate specificity [3]. However, a recent United Kingdom-based randomized controlled trial reported that no significant reduction in ovarian or tubal cancer deaths was observed in the multimodal screening (longitudinal CA125 and second line TVS) or ultrasound screening (TVS first and second-line test) groups compared with the no screening group, which may be attributed to a modest stage-shift of 10–14% [4]. There remains a need for additional circulating marker(s) to improve lead-time detection of disease that would complement the performance shortcomings of CA125.

Using mass spectrometry technology (*see Supplemental Methods),* we assessed the predictive performance of polyamines diacetylspermine (DAS), acetylspermidine (AcSpmd), diacetylspermidine (DiAcSpmd), and N-(3-acetamidopropyl)pyrrolidin-2-one (N3AP) as well as a previously validated 3-marker panel (3MetP: DAS + N3AP + CA125) [5] for detection of OvCa using plasma samples from an NCI-sponsored EDRN reference set consisting of 219 newly diagnosed OvCa cases (59 stage I + II and 160 stage III + IV) as well as 409 healthy controls (Table S1). The 3MetP had an AUC of 0.97 (95% CI: 0.95–0.99) for detection of OvCa, and an AUC of 0.95 (95% CI: 0.91–0.98) when considering early-stage disease (Table S2-4). Among individuals below the clinical cut-off for CA125 (< 35 units/mL), the 3MetP had an AUC of 0.81 (95% CI: 0.70–0.93) (Figure S1).

In our prior study, we demonstrated that, in addition to acetylated polyamines, carbohydrate antigens NANA, NAcMan, and NAcLac as well as the oncometabolite HBA were elevated in plasma of OvCa cases compared to patients with benign pelvic masses [6]. These four metabolites were also found to be significantly (Wilcoxon rank sum test 2-sided *p* < 0.050) elevated in OvCa cases compared to healthy controls with AUC estimates ranging from 0.57–0.91 (Table S2-3).

Using a novel Sensitivity Maximization At A Given Specificity (SMAGS) method (*see Supplemental Methods*), we developed a model consisting of 7 metabolites plus CA125 that yielded an AUC of 0.98 (95% CI: 0.97–0.99) for early-stage disease (Fig. 1A; Table S5). At a 98.5% specificity cutoff, the SMAGS-derived model had sensitivity of 86.2%, correctly identifying 50 of 58 early-stage OvCa cases, which was improved compared to a sensitivity of 75.9% for CA125 alone identifying 44 of 58 early-stage OvCa cases (Table S6). Moreover, the SMAGS-derived model captured 64% of the 14 early stage OvCa cases with CA125 < 35 units/mL, with an AUC estimate of 0.96 (95% CI: 0.92–0.99) (Fig. 1B; Table S7).

**Fig. 1:**
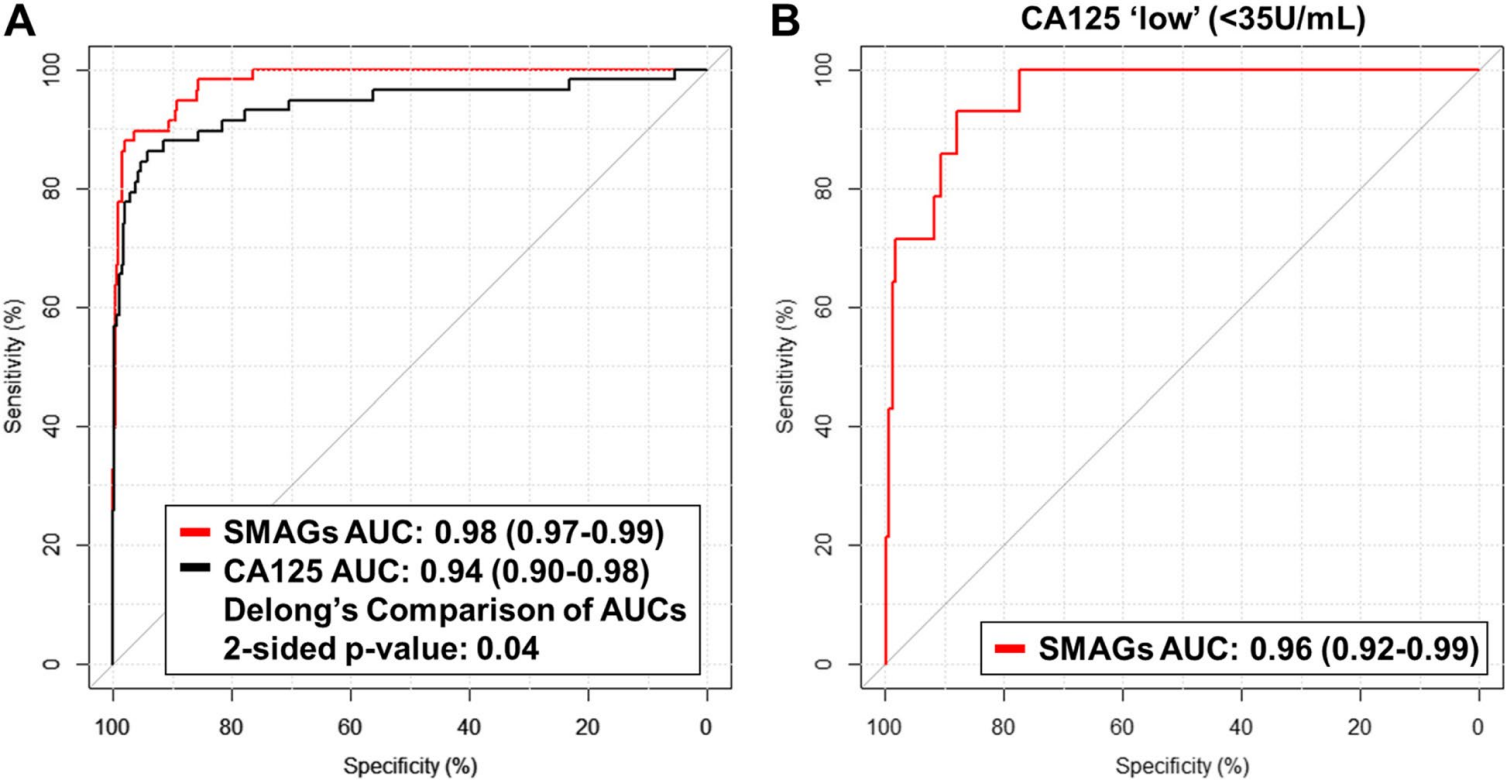
Performance estimates of the SMAGS model for detection of early-stage ovarian cancer in the EDRN Reference Set. **A** AUC curves for the SMAGS model and CA125 for detection of early-stage ovarian cancer. **B** AUC curves for the SMAGS model for detection of early-stage ovarian cancer among individuals with CA125 levels < 35 units/mL

The SMAGS-derived model was validated in an independent set of plasma samples from 65 early stage (I + II) OvCa cases and 141 healthy female controls (Testing Set). The SMAGS-derived model had an AUC of 0.91 (95% CI: 0.87–0.95) for early-stage OvCa, which was improved compared to CA125 alone (AUC: 0.85 (95% CI: 0.78–0.91); 2-sided *p*-value: 0.04) (Fig. 2A; Figure S2). Using the same cut point developed in the EDRN reference set, the SMAGS-derived model achieved a sensitivity of 73.8% and specificity of 91.4%. In comparison, CA125 at the clinical cutoff of 35 units/mL had 55.4% sensitivity and specificity of 97.2% (Tables S6).

**Fig. 2:**
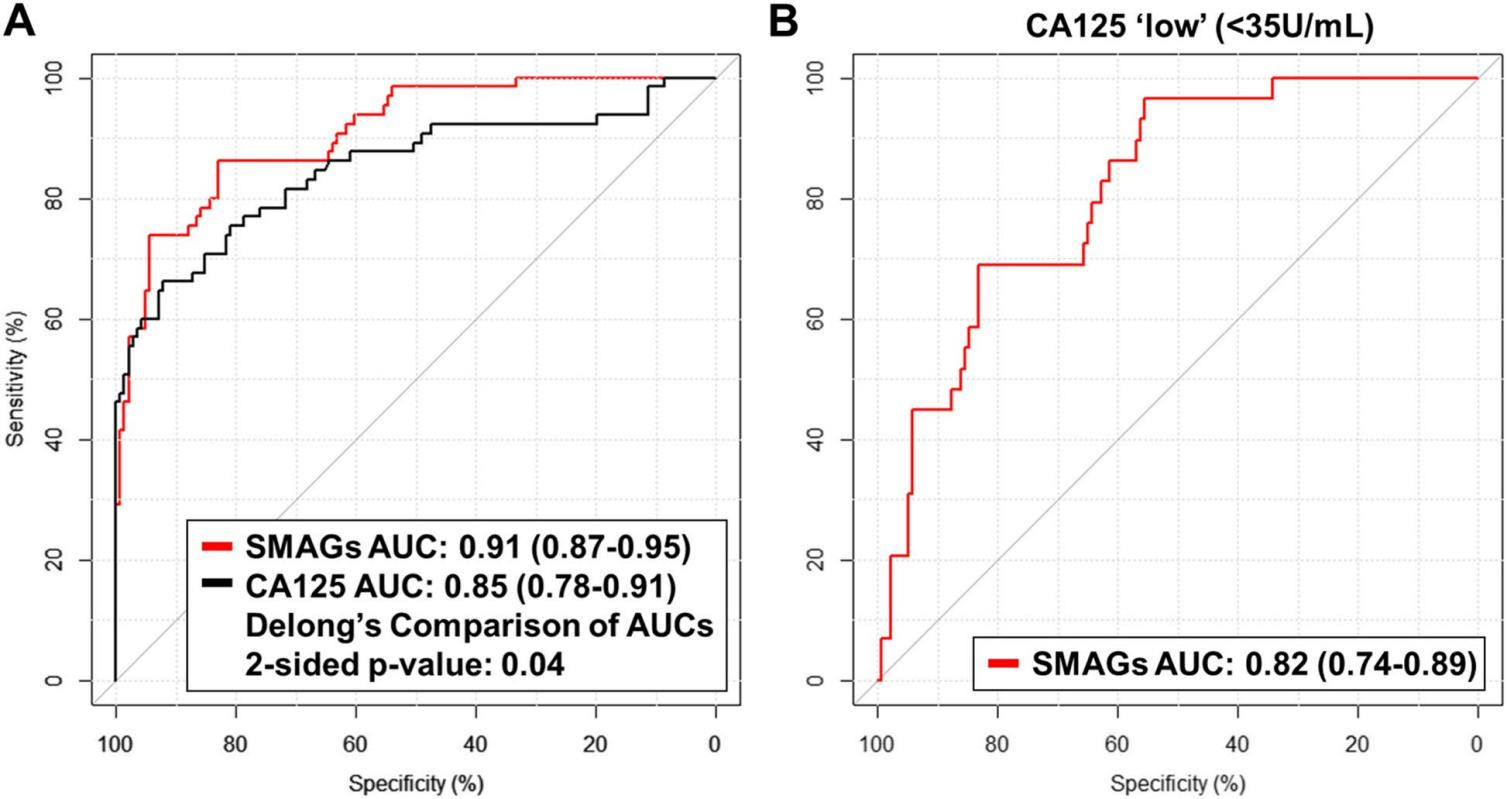
Performance estimates of the SMAGs model for detection of early-stage ovarian cancer in the independent Test Set. **A** AUC curves for the SMAGs model and CA125 for detection of early-stage ovarian cancer. **B** AUC curves for the SMAGs model for detection of early-stage ovarian cancer among individuals with CA125 levels < 35 units/mL

Among the 29 early-stage OvCa cases with CA125 levels < 35 units/mL, the SMAGS-derived model had an AUC of 0.82 (95% CI: 0.74–0.89) (Fig. 2). At the cut point, the SMAGS-derived captured 13 of the 28 early-stage OvCa cases (44.8% sensitivity) that would otherwise have been missed by CA125 (Tables S7).

The blood-based metabolite test provides a potential clinical tool for identifying women at high-risk of harboring OvCa and that would benefit from surveillance and screening with TSV or MRI for earlier detection of disease, which is anticipated to result in mortality reduction due to OvCa [7, 8]. Given the low incidence of ovarian cancer (11.4 in every 100,000 women) in the general population, the blood-based metabolite test may best be suited for detection of OvCa among higher-risk women presenting with non-specific symptoms such as pelvic/abdominal pain [9, 10] or those with BRCA pathological variants [11].

### Supplementary Information

Supplementary Material 1.

## Data Availability

No datasets were generated or analysed during the current study.
