# Supplementary material for: A metabolite-based liquid biopsy for detection of ovarian cancer
Source: Biomark Res. 2024 Aug 28;12:91. doi: 10.1186/s40364-024-00629-2 (PMC11350929; doi:10.1186/s40364-024-00629-2)

**Methods**

***Biospecimens***

In a collaboration sponsored by the NCI Early Detection Research Network (EDRN) blood specimens were obtained preoperatively with informed consent under IRB/ethical committees approved protocols at the University of Texas M.D. Anderson Cancer Center (MDACC, LAB04-0687) and at the Fred Hutchinson Cancer Research Center (FHCRC, IRB 4563).(5) Control plasma were obtained from women who did not develop cancer while participating in the Normal Risk Ovarian Screening Study (NROSS) trial coordinated by MDACC(6) or were healthy donors at the FHCC. All plasma samples were processed and separated with standard operating procedures on the day that blood was obtained and registered in MDACC’s Tissue Station with basic information regarding trial, age, stage, histotype and grade. Aliquots were frozen and stored at -80°C. All participants had provided consent for use of samples in ethically approved secondary studies. The EDRN reference set consisted of 219 newly diagnosed OvCa cases (59 stage I+II and 160 stage III+IV) as well as 409 healthy controls and detailed information is provided in **Table 1**.

For the test set, we leveraged metabolomic profiles previously generated on an independent set of plasma samples from 61 early-stage OvCa cases procured from the MD Anderson Gynecologic Cancer Bank and 141 control samples from the NROSS study(7); detailed information is provided in **Table 1**. All biospecimen were processed at a central site using a standardized protocol; EDTA-plasmas were stored in -80°C until use. Ethical approval was obtained for these samples from the appropriate institutional review boards/ethic committees at MD Anderson and collaborating institutions. All participants had consent for the use of samples in ethically approved secondary studies.

BD vacutainer EDTA tubes (plasma) are spun down at 2500 RPM (1245 G) for 10 minutes at 4°C and processed within 24 hours of collection date.

***CA125 Assays***

CA125 II was assayed on the Roche ELecsys Cobas platform (Roche, Indianapolis, IN) in an MDACC Department of Pathology research laboratory under CLIA conditions. All assays were performed according to the manufacturer’s instructions. The values of >35 U/mL for CA125II were considered positive.

***Metabolomic analyses***

Measurement of plasma acetylated polyamines, carbohydrate antigens, and HIBA was conducted on a Waters Acquity™ UPLC system with 2D column regeneration (I-class and H-class) coupled to a Xevo G2-XS quadrupole time-of-flight (qTOF) mass spectrometer as described in our prior study.(7) Mass spectrometry data was acquired in sensitivity, positive electrospray ionization mode. Acquisition was carried out with instrument auto-gain control to optimize sensitivity during sample acquisition.(8, 9)

Peak picking and retention time alignment of LC-MS and MSe data were performed using Progenesis QI software (Nonlinear, Waters). Data processing and peak annotations were performed using an in-house automated pipeline. Annotations for polyamines were determined by matching accurate mass and retention times using customized libraries created from authentic standards and by matching experimental tandem mass spectrometry data against the NIST MSMS, LipidBlast or HMDB v3 theoretical fragmentations. To correct for injection order drift, each feature was normalized using data from repeat injections of quality control samples collected every 10 injections throughout the run sequence. Measurement data were smoothed by Locally Weighted Scatterplot Smoothing (LOESS) signal correction (QC-RLSC) as previously described.(8, 9)

***Statistical Analyses***

Details regarding the SMAGs model as well as the corresponding program language are provided in Github (https://github.com/smahmoodghasemi/SMAGS). The SMAGs model calculates the probability of an observation belonging to a particular outcome class by estimating the optimum linear combination of the predictors with the intention of maximizing sensitivity at a given specificity. Therefore, if we consider k features ($F_{1}$,.., $F_{k}$), the intention is to find the optimum coefficients ($\beta_{0},\beta_{1}, \ldots, \beta_{k}$) in which the sigmoid function of the linear combination for obersvation i, ($L_{i}$), yields the highest sensitivity at a fixed specificity. Therefore we have:

$$L_{i}=\beta_{1}F_{i1}+\ldots+\beta_{k}F_{ik}+\beta_{0}$$

$$O_{i}=sigmoid(L_{i})$$

$$\hat{y}_{i}=\left\{ \begin{aligned} \begin{matrix} 0 & if O_{i}\leq T \end{matrix} \\ \begin{matrix} 1 & if O_{i}>T \end{matrix} \end{aligned} \right.$$

where $T$ is the threshold calculated by the predefined specificity: $T=quantile \left( \left[ O_{1},\ldots,O_{n} \right], SP \right)$

If we define $\hat{\boldsymbol{y}}$ as the predicted value and $\boldsymbol{y}$ as the observed value, then sensitivity is $P\left( \hat{\boldsymbol{y}}=1 \right|\boldsymbol{y}=1)$ (the proportion of case samples which are correctly classified as cases) and specificity is $1- P\left( \hat{\boldsymbol{y}}=1 \right|\boldsymbol{y}=0)$ (the proportion of control samples which are correctly classified as control). Thus, our optimization function is:

$$Max P\left( \hat{\boldsymbol{y}}=1 \right|\boldsymbol{y}=1)$$

$$Subject to 1- P\left( \hat{\boldsymbol{y}}=1 \right|\boldsymbol{y}=0)=SP$$

Here, $SP$ is the specificity threshold that we set based on the clinical need. In the optimization process, a diverse array of optimization techniques is used to determine the optimal coefficients and intercept values including “Nelder-Mead”, (10)“Powell”, (11)“Conjugate Gradient”, (12)“quasi-Newton method of Broyden, Fletcher, Goldfarb, and Shanno (BFGS)”, (12)“L- BFGS-B”, (13)“TNC”, (14)“Constrained Optimization BY Linear Approximation (COBYLA)”, (15)“Sequential Least Squares Programming (SLSQP)” (16)and “trust-region algorithm for constrained optimization”. (12)The initial values for the optimization algorithm derived from logistic regression.

Receiver operating characteristic curves were generated using R (R version 4.3.1). The 95% confidence intervals presented for individual performance of each biomarker were based on a bootstrap procedure in which we re-sampled with replacement separately for the controls and the diseased 1,000 bootstrap samples.

**Table S1. Patient and tumor characteristics.**

|  | **EDRN Reference Set** | | **Independent Test Set** | |
| --- | --- | --- | --- | --- |
| **Variable** | **Cases** | **Controls** | **Cases** | **Controls** |
| N | 219 | 409 | 65 | 141 |
| age, median (IQR) | 61 (53-70) | 66 (62-70) | 56 (44-67) | 67 (61-72) |
| Stage, N (%) |  |  |  |  |
| Early-stage | 59 (27) | - | 65 (102) | - |
| Late-stage | 160 (73) | - | - | - |
| Histological  Subtype, N (%) |  |  |  |  |
| Serous | 183 (84) | - | 22 (34) | - |
| Non-serous | 32 (15) | - | 43 (67) | - |
| Unknown | 3 (1) | - | - | - |
| CA125 (U/mL)  (median [IQR]) | 292.5  (94.0-756.9) | 11.4  (9.1-16.1) | 425.5  (17.4-211.1) | 11.5  (8.4-16.1) |

Abbreviations: IQR-interquartile range

**Table S2. Performance estimates of individual metabolite biomarkers for detection of Ovarian Cancer in the EDRN Reference Set.**

| **Comparison** | **N, Cases** | **N, Controls** | **N3AP†** | **AcSpmd†** | **DiAcSpmd†** | **DAS†** | **NANA†** | **NAcMan†** | **NAcLac†** | **HIBA†** |
| --- | --- | --- | --- | --- | --- | --- | --- | --- | --- | --- |
| Cases vs Controls | 216 | 409 | 0.62  (0.57-0.66) | 0.75  (0.71-0.79) | 0.73  (0.68-0.77) | 0.92  (0.90-0.95) | 0.62  (0.57-0.66) | 0.57  (0.52-0.62) | 0.61  (0.56-0.66) | 0.91  (0.89-0.94) |
| Early-Stage Cases vs Controls | 59 | 409 | 0.60  (0.52-0.68) | 0.70  (0.62-0.77) | 0.68  (0.61-0.75) | 0.88  (0.83-0.93) | 0.52  (0.43-0.60) | 0.55  (0.47-0.63) | 0.55  (0.47-0.64) | 0.90  (0.85-0.94) |
| Late-Stage Cases vs Controls | 160 | 409 | 0.62  (0.56-0.67) | 0.78  (0.73-0.82) | 0.73  (0.69-0.78) | 0.94  (0.91-0.96) | 0.65  (0.60-0.70) | 0.57  (0.52-0.63) | 0.63  (0.58-0.69) | 0.92  (0.89-0.95) |
| Serous vs Controls | 183 | 409 | 0.62  (0.57-0.66) | 0.75  (0.71-0.80) | 0.72  (0.67-0.76) | 0.93  (0.90-0.95) | 0.62  (0.57-0.67) | 0.57  (0.52-0.63) | 0.64  (0.59-0.69) | 0.91  (0.88-0.94) |
| Non-serous vs Controls | 33 | 409 | 0.60  (0.50-0.70) | 0.76  (0.67-0.85) | 0.76  (0.68-0.83) | 0.89  (0.83-0.95) | 0.60  (0.48-0.71) | 0.54  (0.44-0.64) | 0.47  (0.36-0.58) | 0.94  (0.90-0.99) |

† AUC (95% Confidence Interval)

**Table S3. Predictive performance estimates of individual metabolite biomarkers and the SMAGs model for detection of Ovarian Cancer in the EDRN Reference Set stratified by collection site.**

| **MDACC** | | | | | | | | | | | |
| --- | --- | --- | --- | --- | --- | --- | --- | --- | --- | --- | --- |
| **Comparison** | **N, Cases** | **N, Controls** | **N3AP†** | **AcSpmd†** | **DiAcSpmd†** | **DAS†** | **NANA†** | **NAcMan†** | **NAcLac†** | **HIBA†** | **SMAGs†** |
| Cases vs Controls | 118 | 132 | 0.53  (0.45-0.60) | 0.65  (0.58-0.72) | 0.66  (0.60-0.73) | 0.90  (0.86-0.94) | 0.54  (0.46-0.61) | 0.53  (0.46-0.61) | 0.65  (0.58-0.72) | 0.84  (0.79-0.89) | 0.97  (0.96-0.99) |
| Early-Stage Cases vs Controls | 20 | 132 | 0.59  (0.45-0.72) | 0.61  (0.49-0.74) | 0.60  (0.48-0.73) | 0.87  (0.78-0.96) | 0.45  (0.33-0.58) | 0.63  (0.50-0.77) | 0.64  (0.49-0.79) | 0.71  (0.58-0.83) | 0.94  (0.89-0.99) |
| Late-Stage Cases vs Controls | 98 | 132 | 0.51  (0.43-0.59) | 0.66  (0.58-0.73) | 0.68  (0.61-0.75) | 0.91  (0.87-0.94) | 0.55  (0.48-0.63) | 0.51  (0.44-0.59) | 0.65  (0.58-0.73) | 0.86  (0.82-0.91) | 0.98  (0.96-1.00) |
| **FHCC** | | | | | | | | | | | |
| **Comparison** | **N, Cases** | **N, Controls** | **N3AP†** | **AcSpmd†** | **DiAcSpmd†** | **DAS†** | **NANA†** | **NAcMan†** | **NAcLac†** | **HIBA†** | **SMAGs†** |
| Cases vs Controls | 101 | 277 | 0.63  (0.57-0.69) | 0.81  (0.76-0.86) | 0.74  (0.68-0.80) | 0.93  (0.90-0.96) | 0.64  (0.58-0.70) | 0.54  (0.47-0.61) | 0.52  (0.46-0.59) | 0.97  (0.94-0.99) | 1.00  (0.99-1.00) |
| Early-Stage Cases vs Controls | 39 | 277 | 0.61  (0.52-0.70) | 0.74  (0.64-0.83) | 0.73  (0.64-0.82) | 0.89  (0.83-0.95) | 0.54  (0.42-0.65) | 0.52  (0.42-0.61) | 0.52  (0.42-0.62) | 0.98  (0.96-1.00) | 0.99  (0.99-1.00) |
| Late-Stage Cases vs Controls | 62 | 277 | 0.64  (0.56-0.72) | 0.86  (0.81-0.91) | 0.76  (0.69-0.83) | 0.95  (0.92-0.99) | 0.70  (0.62-0.77) | 0.55  (0.47-0.64) | 0.53  (0.44-0.61) | 0.96  (0.93-0.99) | 1.00  (0.99-1.00) |

† AUC (95% Confidence Interval)

**Table S4. Predictive performance estimates of the 3MetP for detection of ovarian cancer in the EDRN Reference Set.**

|  | **EDRN Reference Set (MDACC + FHCC)** | | | |
| --- | --- | --- | --- | --- |
| **Group** | **Cases, N** | **Controls, N** | **AUC**  **(95% CI)** | **Sensitivity**  **@ 98.5% Specificity** |
| Cases vs Controls | 216 | 409 | 0.97 (0.95-0.99) | 72.7 |
| Early-Stage Cases vs Controls | 59 | 409 | 0.95 (0.91-0.98) | 44.1 |
| Late-Stage Cases vs Controls | 157 | 409 | 0.98 (0.96-0.99) | 83.4 |
| Serous vs Controls | 180 | 409 | 0.97 (0.95-0.99) | 76.1 |
| Non-serous vs Controls | 33 | 409 | 0.97 (0.96-0.99) | 51.5 |
|  | **MDACC** | | | |
| **Group** | **Cases, N** | **Controls, N** | **AUC**  **(95% CI)** | **Sensitivity**  **@ 98.5% Specificity** |
| Cases vs Controls | 115 | 132 | 0.96 (0.94-0.99) | 62.6 |
| Early-Stage Cases vs Controls | 20 | 132 | 0.95 (0.91-0.99) | 45.0 |
| Late-Stage Cases vs Controls | 95 | 132 | 0.97 (0.94-0.99) | 66.3 |
| Serous vs Controls | 111 | 132 | 0.96 (0.94-0.99) | 64.0 |
|  | **FHCC** | | | |
| **Group** | **Cases, N** | **Controls, N** | **AUC (95% CI)** | **Sensitivity**  **@ 98.5% Specificity** |
| Cases vs Controls | 101 | 277 | 0.97 (0.95-0.99) | 81.2 |
| Early-Stage Cases vs Controls | 39 | 277 | 0.95 (0.90-1.00) | 64.1 |
| Late-Stage Cases vs Controls | 62 | 277 | 0.98 (0.96-1.00) | 91.9 |
| Serous vs Controls | 69 | 277 | 0.96 (0.93-1.00) | 81.2 |
| Non-serous vs Controls | 32 | 277 | 0.99 (0.98-1.00) | 81.3 |

**Table S5. Predictive performance estimates of the SMAGs model and CA125 in the EDRN reference Set.**

| **Comparison** | **N, Cases** | **N, Controls** | **CA125†** | **SMAGs†** |
| --- | --- | --- | --- | --- |
| Cases vs Controls | 216 | 409 | 0.97 (0.95-0.99) | 0.99 (0.98-1.00) |
| Early-Stage Cases vs Controls | 59 | 409 | 0.93 (0.89-0.98) | 0.98 (0.97-0.99) |
| Late-Stage Cases vs Controls | 157 | 409 | 0.98 (0.97-1.00) | 0.99 (0.98-1.00) |
| Serous Carcinoma vs Controls | 180 | 409 | 0.97 (0.95-0.99) | 0.99 (0.98-1.00) |
| Non-serous vs Controls | 33 | 409 | 0.97 (0.94-1.00) | 0.99 (0.98-1.00) |

† AUC (95% Confidence Interval)

**Table S6. Confusion matrix describing the performance of the classification model corresponding to the SMAGs Model at a 98.5% specificity threshold and CA125 at cutoff of ≥35 units/mL in the EDRN Reference Set and the Independent Test Set.**

|  | **EDRN Reference Set** | | | | | | |
| --- | --- | --- | --- | --- | --- | --- | --- |
|  | **SMAGs Model** | | |  | **CA125 ≥ 35 units/mL** | | |
|  |  | **Actual** | |  |  | **Actual** | |
|  |  | **1-Case** | **0-Control** |  |  | **1-Case** | **0-Control** |
| **Prediction** | **1-Case** | 50 | 6 | **Prediction** | **1-Case** | 44 | 8 |
|  | **0-Control** | 8 | 403 |  | **0-Control** | 14 | 401 |

|  | **Independent Test Set** | | | | | | |
| --- | --- | --- | --- | --- | --- | --- | --- |
|  | **SMAGs Model** | | |  | **CA125 ≥ 35 units/mL** | | |
|  |  | **Actual** | |  |  | **Actual** | |
|  |  | **1-Case** | **0-Control** |  |  | **1-Case** | **0-Control** |
| **Prediction** | **1-Case** | 48 | 12 | **Prediction** | **1-Case** | 36 | 4 |
|  | **0-Control** | 17 | 129 |  | **0-Control** | 29 | 137 |

**Table S7. Confusion matrix describing the performance of the classification model corresponding to the SMAGs Model among individuals with CA125 levels < 35 units/mL in the EDRN Reference Set and the Independent Test Set.**

|  | **EDRN Reference Set** | | |  | **Independent Testing Set** | | |
| --- | --- | --- | --- | --- | --- | --- | --- |
|  |  | **SMAGs Model** | |  |  | **SMAGs Model** | |
|  |  | **Actual** | |  |  | **Actual** | |
|  |  | **1-Case** | **0-Control** |  |  | **1-Case** | **0-Control** |
| **Prediction** | **1-Case** | 9 | 5 | **Prediction** | **1-Case** | 13 | 12 |
|  | **0-Control** | 5 | 396 |  | **0-Control** | 16 | 125 |

**Figure S1. Classifier performance of the 3MetP for detecting ovarian cancer among individuals with CA125 levels <35 units/mL in the EDRN Reference Set.**

**
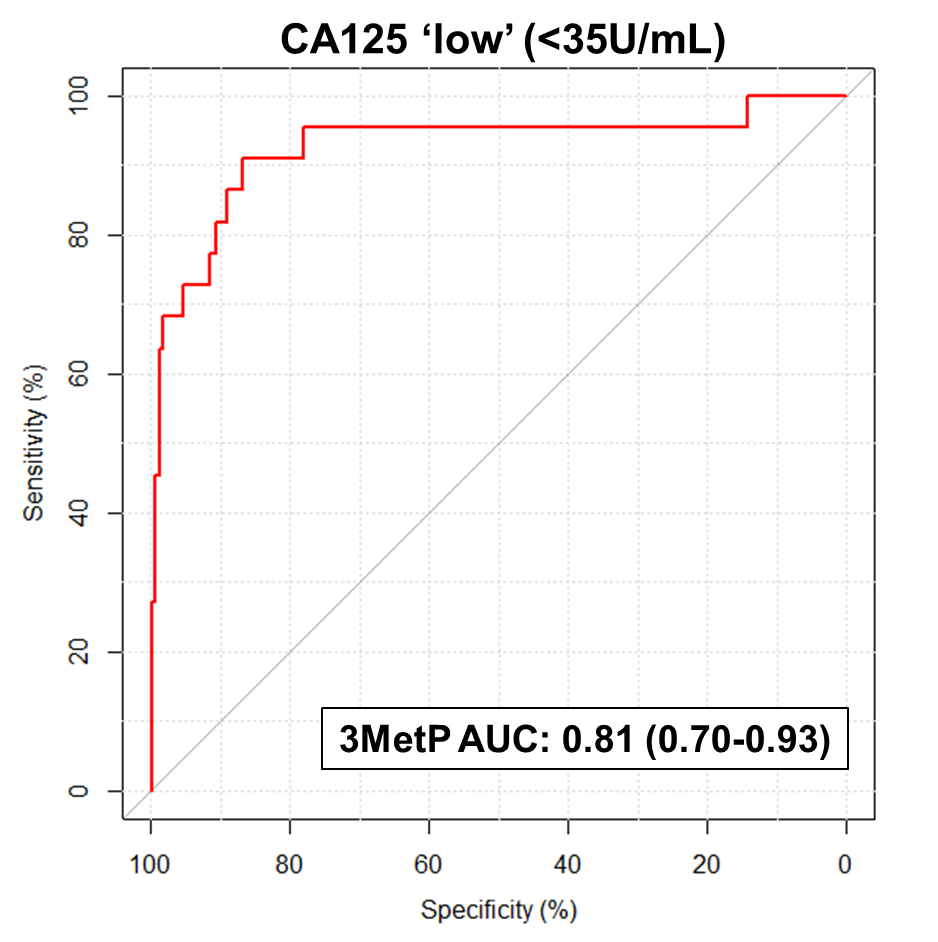
**

**Figure S2. Classifier performance of the SMAGs model and CA125 for distinguishing early-stage ovarian cancers stratified into serous and non-serous from healthy controls in the independent Test Set.**


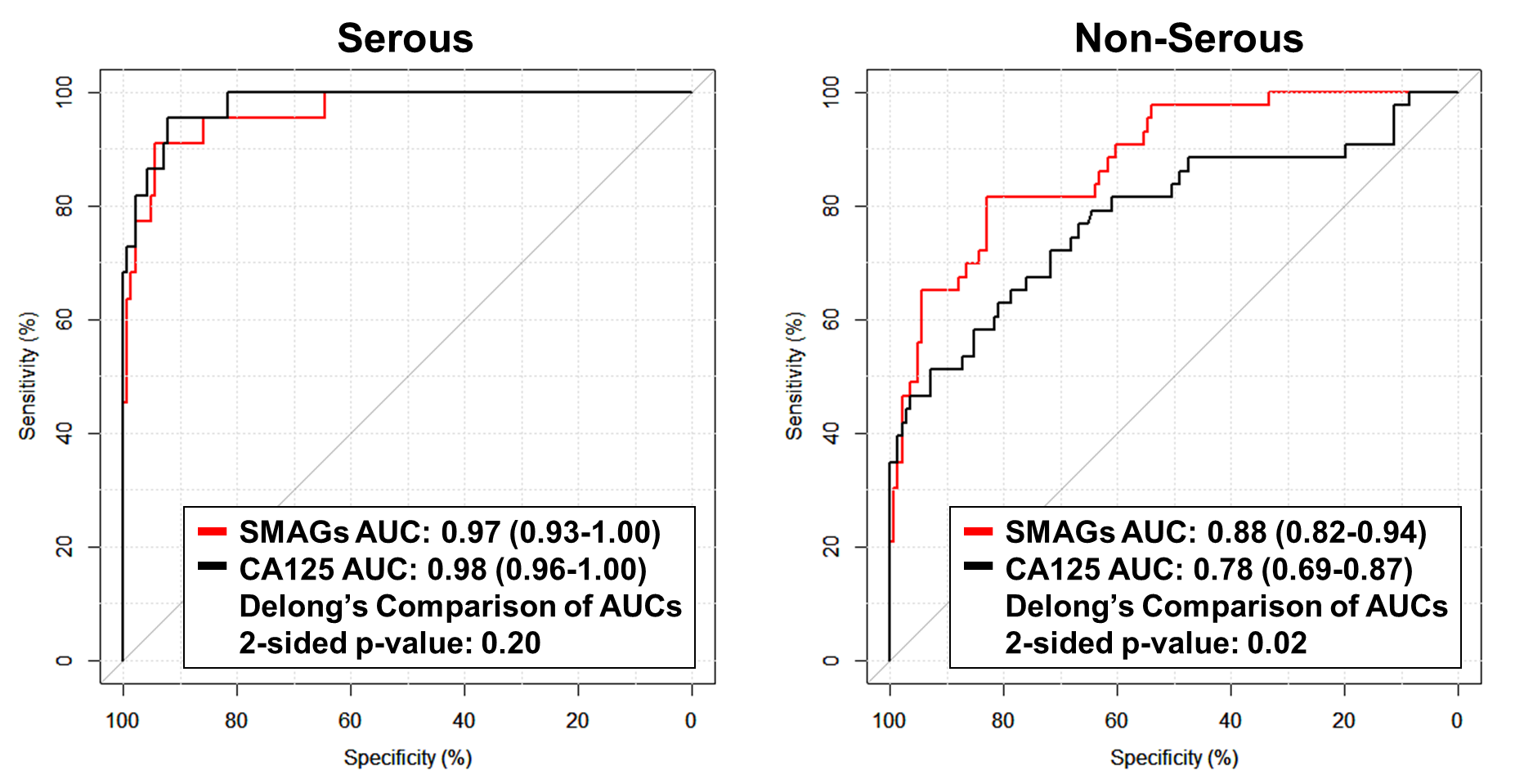

Supplement: Supplementary file 1 — Supplementary Material 1. [file 40364_2024_629_MOESM1_ESM.docx]
